# A Study for Expanding Application Sites for Rotigotine Transdermal Patch

**DOI:** 10.1155/2020/5892163

**Published:** 2020-08-10

**Authors:** Hitoshi Kujirai, Sakiko Itaya, Yumi Ono, Makoto Takahashi, Akira Inaba, Yasushi Shimo, Nobutaka Hattori, Satoshi Orimo

**Affiliations:** ^1^Department of Neurology, Kanto Central Hospital, Tokyo 158-8531, Japan; ^2^Department of Neurology, Juntendo University School of Medicine, Tokyo 113-8421, Japan; ^3^School of Computing, Tokyo Institute of Technology, Tokyo, Kanagawa 226-8503, Japan

## Abstract

The rotigotine transdermal patch (RTP) is a dopamine agonist used to treat Parkinson's disease (PD). Some PD patients cannot continue RTP treatment due to application site reactions. We explored sites for RTP where application site reactions are less severe than those in the six approved application sites. Thirty PD patients (12 men, mean age = 76 years) who underwent RTP at the approved sites and had some application site reactions were enrolled in this study. When applying the RTP to the approved application sites for more than four weeks (pre-RTP) and then on the shin for the following four weeks (post-RTP), skin reactions, itching evaluated using the skin irritation score, motor symptoms, clinical global impressions scale, and plasma rotigotine concentration were examined. The mean visual analogue scale and skin irritation score in the post-RTP group were significantly lower than those in the pre-RTP group. The mean Movement Disorder Society-Unified Parkinson's Disease Rating Scale part III score in the post-RTP group was slightly but significantly lower than that in the pre-RTP group. Plasma rotigotine concentration in the post-RTP group was slightly but significantly lower than that in the pre-RTP group. These results indicate that the shin can be a useful application site for RTP.

## 1. Introduction

Rotigotine is a nonergoline dopamine receptor agonist with D1–5 receptor activity and selective serotonergic and adrenergic activity [1]. The rotigotine transdermal patch (RTP) can maintain stable plasma concentrations over 24 h with a single daily application due to the continuous transdermal delivery [2]. RTP is indicated as a monotherapy for the treatment of early stage Parkinson's disease (PD), as well as a combination therapy with levodopa. RTP has some advantages over other nonergoline dopamine agonists, such as pramipexole and ropinirole, including reduced adverse effects related to gastrointestinal disturbances [3], impulse control disorder [4], and cognitive decline [5, 6]. Currently, there are six approved application sites (abdomen, shoulder, upper arm, hip, thigh, and flank).

RTP is generally well tolerated, with the most common adverse events being application site reactions, gastrointestinal disturbance, somnolence, and headache. Application site reactions have been reported as the most common adverse events in RTP clinical studies. 18% to 44% of patients receiving RTP had application site reactions, including erythema, pruritus, and dermatitis, compared with 11–21% of patients receiving placebo [7, 8]. In addition, 31% of patients receiving RTP for six years showed application site reactions [9]. However, application site reactions were generally mild to moderate in severity and appeared to be dose related. In total, 1–8% of patients receiving RTP withdrew due to application site reactions. On the other hand, in a phase III clinical study conducted in Japan, application site reactions were reported in 97 (57.7%) out of 168 patients [10] and 57 (65.5%) out of 87 patients [11], which are higher rates than those reported in clinical studies from Europe and the United States.

In this study, we aimed to assess the adequate attachment site of RTP where application site reactions are less severe than those in six approved application sites. Then, we focused on the shin because it is very easy for the patients to attach RTP on the shin by themselves.

## 2. Materials and Methods

### 2.1. Study Designs

We did an open-label clinical study to assess the adequate attachment site of RTP where application site reactions are less severe than those in six approved application sites. Then, we focused on the shin because it is very easy for patients to attach RTP on the shin by themselves. The study was conducted at Kanto Central Hospital with the approval of the Kanto Central Hospital ethics committee. Informed consent was obtained from all patients.

### 2.2. Patients

All patients were examined in the outpatient department of Kanto Central Hospital. The inclusion criteria are as follows: patients (aged 50–85 years) were eligible if they had a clinical diagnosis of PD according to the International Parkinson and Movement Disorder Society (MDS) PD clinical diagnostic criteria launched in 2015 [12] more than three years ago. They had to be on stable treatment (no dose change within four weeks before enrolment) with levodopa or stable doses of any concomitant anti-parkinsonian medications. They had to have been receiving RTP at an approved application site for more than four weeks and have some application site reaction. The exclusion criteria are as follows: patients with clinically significant and unstable cardiovascular disease or psychiatric illness including dementia, major depression, and impulse control disorders, or any other medical disorders that might have placed the patients at increased risk were excluded.

### 2.3. Method

The patients underwent RTP at their approved application site for more than four weeks (pre-RTP). The patch was then placed on the shin for the following four weeks (post-RTP). Application site reactions, motor symptoms, motor performance, global effectiveness, and plasma rotigotine concentration were compared between the two periods.

### 2.4. Application Site Reactions

The application site reactions were evaluated using objective and subjective rating scales comprising the skin irritation score and visual analog scale (VAS), respectively. The skin irritation score is divided into six grades as follows: nonirritation (0), minimal irritation (0.5), moderate irritation (1), irritation + edema, papule (2), irritation + papule, small blister (3), and large blister (4). The VAS scale was evaluated by the patients themselves on a scale ranging from no itching (0) to the strongest imaginable itching (100) [13].

### 2.5. Motor Symptoms and Performance

Motor symptoms were evaluated using the MDS-Unified Parkinson's Disease Rating Scale (UPDRS) part III [12], which has 18 items. Each subscale has 0–4 ratings, where 0 = normal, 1 = slight, 2 = mild, 3 = moderate, and 4 = severe. Motor performance was evaluated using timed up and go (TUG) test [14]: the number of the steps and the time required that a person takes to rise from a chair, walk three meters, turn around, walk back to the chair, and sit down. The clinical global impression of improvement (CGI-I) was evaluated by doctors in charge as follows: “0 = not assessed,” “1 = very much improved,” “2 = much improved,” “3 = minimally improved,” “4 = no change,” “5 = minimally worse,” “6 = much worse,” and “7 = very much worse” [15].

### 2.6. Plasma Rotigotine Concentrations

Plasma rotigotine concentration was measured using Liquid Chromatography/Mass Spectrometry (LC-MS/MS). Using 500 mg of human plasma, liquid to liquid extraction with n-hexane was carried out twice under basic conditions and the upper layer (organic layer) was separated. A 2% formic acid solution was added to the separated upper layer and extracted and this was carried out again. Methanol was added to the aqueous layer after acid tolerance to prepare an LC-MS/MS sample. This sample was injected into a reversed-phase high performance liquid chromatography column and several positive ions were measured [16].

### 2.7. Hair on the Shins

This separate substudy was conducted in the outpatient department of Kanto Central Hospital to see whether the shin could be a feasible application site for RTP in 154 consecutive Japanese patients with PD (46–95 ,75.3 years; 72 men). As a result, 132 and 15 out of 154 patients had no and nearly no hair, respectively. This indicates that, in most Japanese patients (95.5%) with PD, the shins can be used to attach RTP.

### 2.8. Statistical Analysis

VAS and skin irritation scores, MDS-UPDRS part III score, number of steps and TUG time in the TUG test, and plasma rotigotine concentrations were expressed as means ± SD. Differences among groups were compared using Wilcoxon rank sum test. A *p* value less than 0.05 was considered significant.

## 3. Results

### 3.1. Patients

Between April 1st, 2017, and November 30th, 2017, Japanese patients with PD were enrolled in this study. The clinical characteristics of the patients are shown in Table 1. The patients included 12 men and 18 women, average age 77.8 ± 5.5 (63–86) years, Hoehn-Yahr stage 3.1 ± 0.7 (stage 2, *n* = 5; stage 3, *n* = 18; stage 4, *n* = 6; and stage 5, *n* = 1), and disease duration of 8.7 ± 3.4 (3–15) years. The mean dosage of RTP was 13.7 ± 6.8 (4.5–31.5) mg, and the levodopa equivalent dose (LED) [17] was 994.8 ± 212.8 (448.5–1546.5) mg.

### 3.2. VAS Scale and Skin Irritation Score

**Figure 1:**
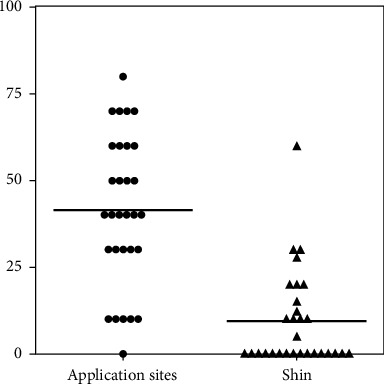
The VAS scale was significantly lower in the post-RTP group than in the pre-RTP group (*p* < 0.01).

**Figure 2:**
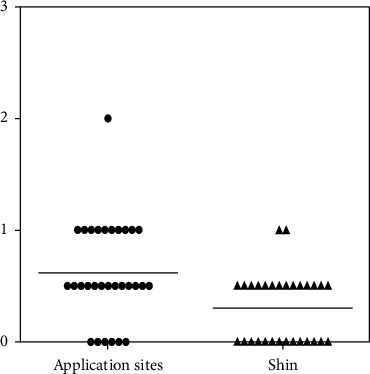
The skin irritation score of the post-RTP group was significantly lower in the pre-RTP group (*p* < 0.05).

The VAS scale was significantly lower in the post-RTP group (9.3 ± 14.0) than in the pre-RTP group (41.3 ± 19.7) (*p*=0.0066, *p* < 0.01) (Figure 1). The VAS scale in the post-RTP group was reduced in 28 patients, increased in one patient, and not changed in one patient. Skin irritation scores were significantly lower in the post-RTP group (0.3 ± 0.4) than in the pre-RTP group (0.6 ± 0.4) (*p*=0.0117, *p* < 0.05) (Figure 2). The skin irritation score of the post-RTP group was reduced in 19 patients, increased in three patients, and not changed in eight patients.

### 3.3. MDS-UPDRS Part III

**Figure 3:**
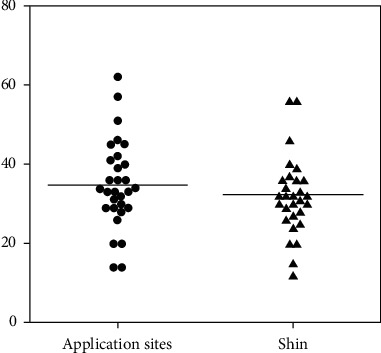
The MDS-UPDRS part III score was significantly lower in the post-RTP group than that in the pre-RTP group (*p* < 0.05).

The MDS-UPDRS part III score was significantly lower in the post-RTP group (32.3 ± 10.2) than that in the pre-RTP group (34.9 ± 11.5) (*p*=0.0167, *p* < 0.05) (Figure 3). However, the difference between the two groups was very small (7.4%). The MDS-UPDRS part III score of the post-RTP group was reduced in 21 patients, increased in seven patients, and not changed in two patients.

### 3.4. TUG Test

Three tests were conducted for each patient and the average result was compared between the two groups. Twenty-four patients performed TUG test completely. The mean number of steps in the TUG test was 18.6 ± 6.3 in the pre-RTP group and 20.2 ± 9.6 in the post-RTP group, respectively. There was no significant difference between the two groups (*p*=0.663). The mean TUG time was 15.2 ± 8.0 sec in the pre-RTP group and 16.3 ± 11.7 sec in the post-RTP group, respectively. There was no significant difference between the two groups (*p*=0.406).

### 3.5. CGI-I Scale

**Figure 4:**
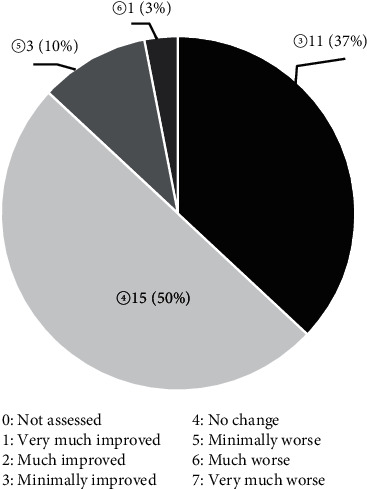
The CGI-I scale in the post-RTP group was minimally improved in 29%, not changed in 50%, minimally worse in 11%, and much worse in 1% relative to pre-RTP.

The CGI-I scale in the post-RTP group was minimally improved in 29%, not changed in 50%, minimally worse in 11%, and much worse in 1% relative to pre-RTP (Figure 4).

### 3.6. Plasma Rotigotine Concentrations

**Figure 5:**
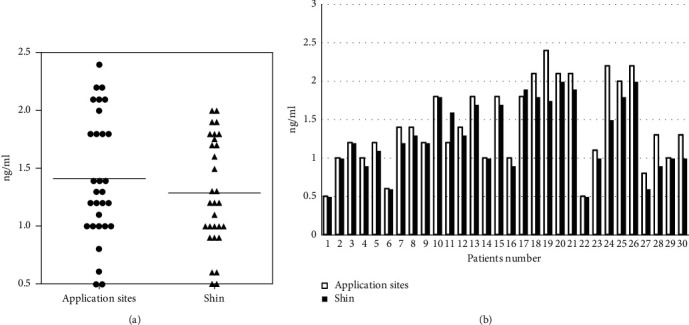
The mean plasma rotigotine concentration in the pre-RTP group was significantly lower than that in the post-RTP group (a) (*p* < 0.005). The plasma rotigotine concentration was reduced in 19, increased in two, and not changed in nine patients (b).

The mean plasma rotigotine concentration in the pre-RTP group (1.288 ± 0.46 [0.5–2.4] ng/ml) was slightly but significantly lower than that in the post-RTP group (1.413 ± 0.53 [0.5–2.0] ng/ml) (*p*=0.0025, *p* < 0.005) (Figure 5(a)). The difference between the two groups was 0.125 ng/mL (9.1%). The plasma rotigotine concentration was reduced in 19 patients, increased in two patients, and not changed in nine patients (Figure 5(b)). In the nine patients with no change in plasma rotigotine concentration, the MDS-UPDRS part III score was reduced in six patients, not changed in one patient, and increased in two patients. In the two patients with increased plasma rotigotine concentration, the MDS-UPDRS part III score was reduced in both patients. In the 19 patients with reduced plasma rotigotine concentration, the MDS-UPDRS part III score was increased in seven and reduced in 12.

## 4. Discussion

In the RTP phase 3 clinical studies in Europe and the United States, the application sites were restricted to the current indication sites because of hair on the chest and shins [18]. A range of 18% to 44% of patients receiving RTP had application site reactions [7–9]. In Japanese patients with PD, application site reactions were present in over 50%, with a slightly higher rate than that seen in Europe [10, 11].

In this study, the application site reactions were significantly reduced in the shin group compared with that seen at the six approved sites group when evaluated using both objective and subjective rating scales. The application site reactions are thought to be induced by the following mechanisms: chemical mediators such as histamine and prostaglandin are released from the mast cells of the skin by external stimulation and these chemical mediators act on the capillaries in the dermis. When the capillaries expand, the skin turns red. Increased capillary permeability is thought to cause plasma components to leak out and cause skin swelling [19]. The application site reactions usually depend on the percutaneous absorption of drugs, which is affected by skin thickness, lipid composition of the stratum corneum, which is the principal barrier to percutaneous drug transport, and differences in the nature of the dermis (number of surface capillaries, sweat and sebaceous glands, diffusivity, and density of skin appendages) [18]. It was reported that the percutaneous absorption rate of drugs was much higher in the head, forehead, lower jaw, axilla, and scrotum than in the forearm, palms, ankles, and soles. This indicates that the percutaneous absorption rate of drugs in distal parts of the body is much lower, resulting in, at least in part, fewer application site reactions.

To date, no studies have investigated the thickness of the stratum corneum in the shin. According to anatomical studies of the number of cells in the stratum corneum, the number of these cells in the distal part of the body, such as the instep and heel, is nearly five times higher than that in the proximal regions of the body, such as the scrotum and abdomen [20]. Because the shin is a distal part of the body, the number of stratum corneum cells in the shin may be higher than that of the approved sites, including the abdomen, shoulder, upper arm, hip, thigh, and flank, resulting in fewer application site reactions.

Plasma rotigotine concentration in the shin group was slightly but significantly reduced compared with that in the approved sites group, which might be due to differences in the percutaneous absorption rate of the drug mentioned above. However, absorption rates of drugs can be changeable, even for the same substance at identical skin sites in different individuals, because individual biological variability is influenced by temperature, skin condition, circulatory effects, and skin metabolism [2]. Rotigotine bioavailability depends on the application site. The area under the curve (AUC) for rotigotine was highest at shoulder application (1.33 ng/mL × h/mg) compared with the upper arm (1.18 ng/mL × h/mg), flank (1.16 ng/mL × h/mg), hip (1.03 ng/mL × h/mg), abdomen (1.01 ng/mL × h/mg), and thigh (0.92 ng/mL × h/mg). The respective mean ratios for AUC ranged between 0.87 (abdomen vs. flank) and 1.46 (shoulder vs. thigh). However, there is no indication of a relevant effect on clinical outcomes [2].

The MDS-UPDRS part III score was slightly but significantly improved in the shin group compared to that in the approved sites group, while the TUG test and CGI-I scores were not different between the two groups, even though the plasma rotigotine concentration in the shin group was slightly but significantly lower than that in the approved sites group. These results suggest that the clinical usefulness of RTP on the shins is at least not inferior to that on the approved sites in patients with PD. We discuss the reason why motor symptoms in the shin group are not inferior to those in the approved sites group, even with the slight reduction in plasma rotigotine concentration in the shin group. The clinically effective plasma concentration of rotigotine is over 0.75 ng/mL [21]. The mean plasma rotigotine concentrations in the shin and approved site groups were 1.288 and 1.413 ng/ml, respectively, which are higher than the clinically effective plasma concentration. The difference between the former and latter was 0.125 ng/ml (9.1%), which is smaller than the difference between the AUC of rotigotine concentration for RTP on the shoulder and thigh [2]. Furthermore, the reduction in the application site reactions may cause a placebo effect, followed by an improvement of motor symptoms. Generally, placebo effects on motor symptoms are prominent in patients with PD [11, 22, 23]. The mechanism of the placebo effect in PD is still unknown, but dopamine release may be involved in this mechanism [24, 25]. Reduction of skin reactions might cause dopamine release, resulting in improvement of motor symptoms.

This study has some limitations. First, this study was an open-label and not double-blinded. MDS-UPDRS III, TUG, and CGI-I may be affected in an open-label study. However, the differences between the shin group and approved sites group were not significant. However, application site reactions can occur regardless of the double-blind or open-label nature of studies. Second, the six application sites (abdomen, shoulder, upper arm, hip, thigh, and flank) and dosages of RTP before RTP on the shins were different between patients. However, we wanted to perform this study in a routine clinical setting. As previously mentioned, even though plasma rotigotine concentrations were different between the six approved sites, there was no indication of a relevant effect on clinical outcomes [2].

## 5. Conclusions

In conclusion, application site reactions as assessed using the VAS scale and skin irritation score in the shin group were significantly improved compared to the approved application site group. MDS-UPDRS part III score was slightly but significantly improved in the shin group compared to that in the approved application site group, although the plasma rotigotine concentration was slightly reduced in the shin group. These results indicate that the shin can be a useful application site for RTP.

## Figures and Tables

**Table 1:** Patients characteristics.

	*N* = 30 (mean ± SD)
M/F	12/18
Age (year of age)	76 ± 5.6
Hoehn-Yahr stage	3.0 ± 0.85
Disease duration (year)	14 ± 4.6
Dosage of rotigotine (mg)	13.65 ± 6.82
LED (levodopa equivalent dosage: mg)	994.83 ± 212.83
MDS-UPDRS part III (approved sites)	35 ± 11.3

## Data Availability

The data used to support the findings of this study have been restricted by the Kanto Central Hospital Ethics Board to protect patient privacy. The data are available from the corresponding authors of researchers who meet the criteria for accessing sensitive data.
